# ROS: Crucial Intermediators in the Pathogenesis of Intervertebral Disc Degeneration

**DOI:** 10.1155/2017/5601593

**Published:** 2017-03-14

**Authors:** Chencheng Feng, Minghui Yang, Minghong Lan, Chang Liu, Yang Zhang, Bo Huang, Huan Liu, Yue Zhou

**Affiliations:** Department of Orthopedics, Xinqiao Hospital, Third Military Medical University, Chongqing 400037, China

## Abstract

Excessive reactive oxygen species (ROS) generation in degenerative intervertebral disc (IVD) indicates the contribution of oxidative stress to IVD degeneration (IDD), giving a novel insight into the pathogenesis of IDD. ROS are crucial intermediators in the signaling network of disc cells. They regulate the matrix metabolism, proinflammatory phenotype, apoptosis, autophagy, and senescence of disc cells. Oxidative stress not only reinforces matrix degradation and inflammation, but also promotes the decrease in the number of viable and functional cells in the microenvironment of IVDs. Moreover, ROS modify matrix proteins in IVDs to cause oxidative damage of disc extracellular matrix, impairing the mechanical function of IVDs. Consequently, the progression of IDD is accelerated. Therefore, a therapeutic strategy targeting oxidative stress would provide a novel perspective for IDD treatment. Various antioxidants have been proposed as effective drugs for IDD treatment. Antioxidant supplementation suppresses ROS production in disc cells to promote the matrix synthesis of disc cells and to prevent disc cells from death and senescence in vitro. However, there is not enough in vivo evidence to support the efficiency of antioxidant supplementation to retard the process of IDD. Further investigations based on in vivo and clinical studies will be required to develop effective antioxidative therapies for IDD.

## 1. Introduction

Intervertebral disc (IVD) degeneration (IDD) is a widely known contributor to low back pain (LBP) that is one of the most prevalent musculoskeletal disorders worldwide and results in a massive socioeconomic burden [1–4]. Degenerative discs show the structural failure that is characterized by disc height collapse, annulus fibrosus (AF) fissures, loss of proteoglycans (PGs) and water in nucleus pulposus (NP), and cartilage endplate (CEP) calcification. IDD is a multifactorial disorder. Its etiological factors include aging, smoking, infection, abnormal mechanical stress, diabetes, trauma, and genetic predisposition [5–11]. The pathogenesis of IDD involves a complex signaling network and various effector molecules [12, 13]. However, our understanding of the pathogenesis of IDD is limited. Elucidating the molecular mechanism of IDD in detail will contribute to developing new measures for the prevention and treatment of IDD.

Recent studies have reported that the establishment and progression of IDD are tightly associated with reactive oxygen species (ROS) and oxidative stress [14–16]. Although the roles of ROS and oxidative stress in various diseases have been widely investigated, including cardiovascular diseases, diabetes, and osteoarthritis [17–19], little attention has been paid to the effect of oxidative stress on the structure and function of IVDs until now. This review provides an overview of the involvement of oxidative stress in the pathogenesis of IDD. ROS are essential mediators of the occurrence and progression of IDD. Thus, antioxidation has been proposed as a promising therapeutic strategy of IDD. The abbreviations used in this review are listed in the Abbreviations section.

## 2. The Microenvironment of Healthy IVDs

The IVD is composed of three distinct anatomical regions, including the central NP, the peripheral AF enclosing the NP, and the CEPs located superiorly and inferiorly. The IVD is the largest avascular structure in human body [45]. Although the cells located at outer AF take nutrients and eliminate metabolites through the capillaries in the soft tissues surrounding IVDs, the nutrient-metabolite homeostasis in discs mainly depends on the exchange of nutrient solutes and metabolites between the capillaries in the adjacent vertebral bodies and the remaining disc cells (inner AF cells, NP cells, and CEP cells) via the diffusion pathway constituted by CEPs and the dense extracellular matrix (ECM) of NP and AF [46, 47]. Therefore, the concentration of oxygen, glucose, and some other nutrients in discs is low while the concentration of metabolites is high. The microenvironment of healthy discs is characterized by hypoxia (1-2% O_2_), low nutrition, and acidic PH due to lactic acid accumulation [48].

## 3. ROS Production in Healthy IVDs

ROS are a family of unstable and highly reactive molecules with or without free radicals, including superoxide anion (O_2_^−^), hydroxyl radical (OH^−^), hydrogen peroxide (H_2_O_2_), and hypochlorite ion (OCl^−^). Also, reactive nitrogen species, such as nitric oxide (NO), are regarded as a member of the ROS superfamily due to their similar effects to ROS. ROS are inevitably produced through the oxygen-using metabolic processes of cells. In other words, ROS production is a price paid for the aerobic metabolism. While the microenvironment of IVDs is characterized by hypoxia due to poor vascularization, all resident disc cells (NP cells, AF cells, and CEP cells) have been demonstrated to be not anaerobic and to have oxygen-utilizing metabolic processes in vivo [49, 50]. Therefore, disc cells are expected to produce ROS in the microenvironment of discs. Actually, H_2_O_2_ has been identified in human NP tissues [51]. Peroxisomes have been detected in human AF cells in vitro [52], suggesting that disc cells are the ROS generator in the microenvironment of discs.

**Figure 1:**
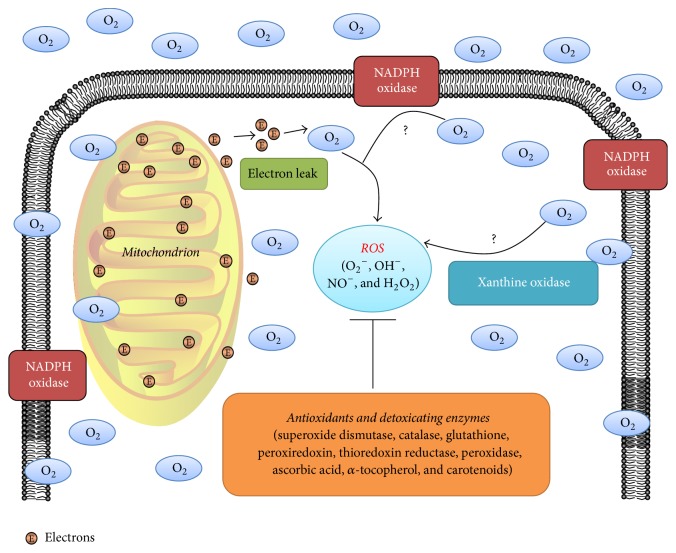
The redox homeostasis of intervertebral disc (IVD) cells. The role of the mitochondrion in reactive oxygen species (ROS) generation of disc cells has been well established. During the transportation of electrons, a small proportion of electrons (1%–3%) leak to produce ROS. However, the nonmitochondrial ROS generation through nicotinamide adenine dinucleotide phosphate (NADPH) oxidase or xanthine oxidase in disc cells remains unknown. Thus, “?” is labeled in these pathways. ROS scavenging is performed by antioxidants and detoxicating enzymes.

Mitochondrion is a major site of ROS generation. During the transportation of electrons, a small proportion of electrons (1%–3%) leak and reduce O_2_ to O_2_^−^ rather than H_2_O [53, 54]. The mitochondrion-dependent ROS production has been reported in various disc cells derived from different species (Figure 1), including human NP cells, human AF cells, rat AF cells, rat notochordal cells, and rabbit NP cells [55–60]. Nonmitochondrial oxygen consumption through nicotinamide adenine dinucleotide phosphate (NADPH) oxidase (NOX) or xanthine oxidase (XO) is one other main ROS production site (Figure 1) [61, 62]. NOX consists of a catalytic subunit gp91^phox^ and its partner p22^phox^ as well as three regulatory subunits (p40^phox^, p47^phox^, and p67^phox^). NOX is a professional ROS-generating enzyme that is responsible for the respiratory burst and phagocytosis of phagocytic cells. Besides, NOX is also expressed by nonphagocytic cells [63]. XO oxidizes hypoxanthine to xanthine to generate H_2_O_2_. The nonmitochondrial oxidative stress has been documented in various diseases, including cardiovascular diseases, lung injury, and central nervous system diseases [17, 62, 64]. However, the expression of NOX and XO in disc cells remains unknown (Figure 1). The nonmitochondrial ROS production of disc cells should be investigated in further studies.

The intracellular redox homeostasis depends on a balance between ROS generation and ROS scavenging performed by nonenzymatic and enzymatic antioxidants (Figure 1), including glutathione (GSH), superoxide dismutase (SOD), catalase (CAT), glutathione peroxidase, ascorbic acid (vitamin C), *α*-tocopherol (vitamin E), and carotenoids [65, 66]. The disturbance of this balance causes oxidative stress that is detrimental to the function and viability of cells [65, 67].

## 4. ROS: Critical Signaling Molecules in Disc Cells

ROS serve as signaling messengers in various signaling pathways, including the nuclear factor-*κ*B (NF-*κ*B) pathway, the mitogen-activated protein kinases (MAPKs) pathway, and the lipid pathways (phospholipases, protein kinase C (PKC), and the phosphatidylinositol-3-kinase (PI3K)/Akt pathway) [68, 69]. However, the signaling response to ROS is cell-type-dependent. In human and bovine NP cells, ROS activated signaling molecules, such as p38, ERKs, JNKs, p65, Akt, and Nrf2, to induce the upregulation of ECM proteases and proinflammatory genes along with the downregulation of ECM genes and anticatabolic genes [15, 20, 21, 57]. Similarly, ROS activated the MAPKs pathway to induce the autophagy of rat NP cells [22] and to regulate the expression of tumor necrosis factor-alpha (TNF-alpha), matrix metalloprotease-3 (MMP-3), cyclooxygenase-2 (COX-2), and aggrecan in rat AF cells [14]. Thus, it can be speculated that ROS regulate the phenotype of disc cells through a complicated signaling network. ROS-sensitive signaling molecules in disc cells are summarized in Table 1. However, our understating of the function of ROS in disc cells is limited. More signaling pathways regulated by ROS in disc cells are required to be elucidated further in depth.

## 5. Disturbed Redox Homeostasis in the Microenvironment of Degenerative IVDs

### 5.1. Excessive ROS Production in Degenerative Discs

**Figure 2:**
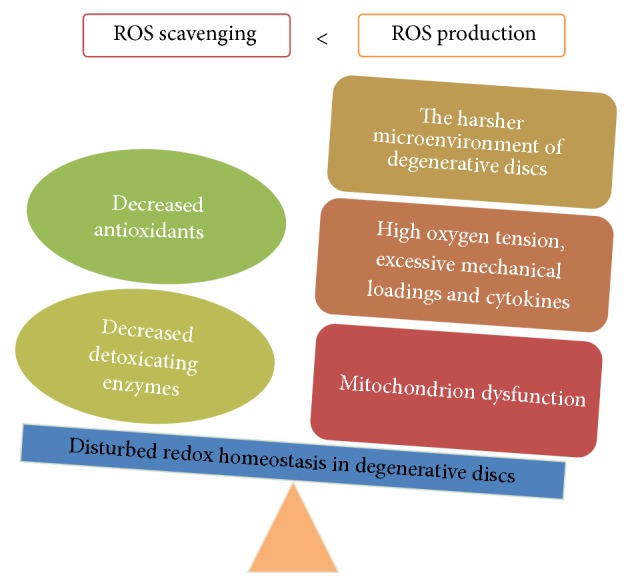
Disturbed redox homeostasis in the microenvironment of degenerative IVDs. Excessive reactive oxygen species (ROS) production and impaired antioxidative system exist in degenerative discs.

In fact, excessive ROS production in degenerative discs has been reported. The level of NO in rat degenerative discs was shown to increase dramatically [16]. Peroxynitrite, a potent oxidative agent derived from the interaction of O_2_^−^ and NO in vivo, can cause tyrosine nitrosylation that is a marker of excessive ROS production [70]. Noticeably, tyrosine nitrosylation was identified in human NP specimens. The percentage of nitrotyrosine-positive cells in human NP tissues increased with IDD advancing [14, 20]. In summary, ROS production in discs increases with IDD progression. With respect to the trigger of excessive ROS production in discs, it is attributed to the harsh microenvironment of degenerative discs in which the availability of nutrients to disc cells and the clearance of metabolites are markedly suppressed due to CEP calcification [46, 71]. In this microenvironment, various exogenous stimuli, such as mechanical loading, high oxygen tension, high glucose stress, and proinflammatory cytokines, increase ROS production in disc cells (Figure 2). Furthermore, ROS themselves also enhance ROS production in disc cells, forming a positive feedback loop [14, 15, 20–24, 36, 44, 56–60].

Mitochondrion dysfunction is a main cause of excessive ROS production (Figure 2). It is characterized by loss of mitochondrial mass, respiratory chain defect, opening of mitochondrial permeability transition pore (MPTP), and decreased mitochondrial membrane potential (MTMP). Dysfunctional mitochondrion leaks more electrons to produce ROS. Previous studies have reported the decreased mitochondrial mass and the reinforced mitochondria respiration in human AF cells with IDD progression. Also, mitochondrion dysfunction-associated genes, such as BCL2-like 11, mitochondrion-associated 1, and programmed cell death 6, were shown to be upregulated in human AF cells isolated from degenerative discs [55, 72]. Structurally, the mitochondrion of human AF cells from degenerative discs showed an abnormal morphology with small cristae, dark colour, and dense inclusion bodies [55]. Moreover, various stimuli have been shown to cause mitochondrion dysfunction in human, rat, and rabbit disc cells, including high oxygen tension, high glucose stress, and abnormal mechanical loading [56–60]. Compression induced the opening of MPTP and decreased MTMP in rabbit NP cells [56]. High glucose disrupted the MTMP of rat disc cells [58–60]. Interestingly, these stimuli also are risk factors of IDD, suggesting that mitochondrion dysfunction is involved in the pathogenesis of IDD. On the other hand, mitochondrion is a primary attack target of ROS. Mitochondrial DNA and respiratory enzymes undergoing oxidative damage cause mitochondrion dysfunction further. As a result, a vicious cycle is formed [22, 73]. A better understanding of the essential role of mitochondrion dysfunction in the establishment and progression of IDD will give a novel insight into the pathogenesis of IDD. Moreover, the contribution of NOX and XO to ROS overproduction in degenerative discs remains to be elucidated.

### 5.2. Antioxidant Decline in Degenerative Discs

Our knowledge about antioxidants in degenerative discs is limited. The activity of SOD in rat lumber discs declined with IDD advancing [16]. Methionine sulfoxide reductase (Msr), a repair enzyme scavenging ROS through reducing methionine residues in oxidation proteins, was downregulated in human senescent AF cells [72], which makes disc cells more susceptive to oxidative damage. These limited lines of evidence suggest an antioxidant decline in degenerative discs that results in the accumulation of ROS in degenerative discs (Figure 2). However, more investigations should be performed to obtain an elaborate picture of the antioxidant status in degenerative discs.

### 5.3. Oxidative Stress in Degenerative Discs

As mentioned above, a disturbed balance between ROS generation and ROS scavenging has been identified in degenerative discs (Figure 2). Therefore, oxidative stress is aroused in the microenvironment of degenerative discs. ROS are able to cause the oxidative damage of DNA, lipids, and proteins. Concomitantly, the by-products of oxidative stress accumulate in degenerative discs. Malondialdehyde (MDA), a product of the peroxidation of polyunsaturated fatty acid residues, was shown to accumulate in rat degenerative discs [16]. Advanced glycation end products (AGEs) are the products of oxidative modifications of glycated proteins, including carboxymethyl-lysine (CML) and pentosidine. They are a marker of oxidative stress [74]. Noticeably, CML and pentosidine were found to accumulate in human degenerative discs, and the level of CML in discs was positively correlated with the degree of IDD [75–77]. Moreover, the levels of carbonylated proteins and advanced oxidation protein products (AOPP) increased significantly in rat and mouse degenerative discs. The protein constituents of discs from aging mice contained more oxidized amino acids than those from young mice [16, 78]. Interestingly, IDD was also reported to be associated with systemic oxidative stress. In the plasma of patients or rats with IDD, SOD activity decreased markedly, and the level of several oxidative stress biomarkers increased dramatically, including phospholipase A, fructoselysine, MDA, peroxidation potential (PP), total hydroperoxides, AOPP, and NO [16, 79]. In short, both systemic oxidative stress and local oxidative stress are reinforced during the process of IDD, indicating the critical role of oxidative stress in the pathogenesis of IDD.

## 6. The Roles of Oxidative Stress in the Pathogenesis of IDD

### 6.1. ROS and Disc Cell Apoptosis

Apoptosis is a programmed cell death characterized by apoptotic body formation, DNA fragmentation, chromosomal condensation, and caspase activation. Apoptosis decreases the number of functional and viable disc cells, which is one of the triggers of IDD [80, 81]. ROS have been determined as a potent proapoptotic factor for human, rat, and rabbit NP cells in vitro [38, 40, 41, 44]. H_2_O_2_ increased lysosome membrane permeability and decreased MTMP in rat NP cells. As a result, ROS were overproduced to induce NP cell apoptosis through the mitochondrial apoptosis pathway [22, 41]. Notochordal cell apoptosis is recognized as the starting point of IDD. Noticeably, H_2_O_2_ induced the apoptosis of rat notochordal cells via both the mitochondrial apoptosis pathway and the death receptor pathway [82]. Besides, ROS mediate the proapoptotic effect of various external stimuli on disc cells, including mechanical loading, nutrition deprivation, proinflammatory cytokines, and local anesthetics (LAs). These stimuli promoted ROS production in rabbit NP and AF cells to induce apoptosis through the mitochondrial apoptosis pathway [23, 56]. Based on the findings, antioxidation is proposed as a potential measure to prevent disc cell apoptosis and to increase the number of functional and viable cells in discs.

### 6.2. ROS and Disc Cell Autophagy

Autophagy is characterized by autophagosome formation. It is a lysosomal catabolism that degrades dysfunctional organelles and damaged proteins to provide recycled metabolic substrates. Thus, autophagy provides energy through self-digestion to protect cells from various external stresses. Recent studies have demonstrated the presence of autophagy in rat and human degenerative disc cells [26, 83, 84]. Noticeably, ROS are a crucial regulator of disc cell autophagy in vitro. H_2_O_2_ promoted the autophagy of rat NP cells via the ERK/mTOR signaling pathway [22]. ROS overproduction induced by mechanical compression was involved in the compression-induced autophagy of rat NP cells [25]. High glucose stress increased ROS production to upregulate the expression of autophagy-related genes in rat notochordal cells [58]. Furthermore, excessive ROS production caused by serum deprivation reinforced the autophagy of rat NP cells through the AMPK/mTOR signaling pathway [24]. However, more lines of evidence based on in vivo studies and human disc cells are still needed to elucidate the role of ROS in regulating disc cell autophagy. With regard to the roles of autophagy in the pathogenesis of IDD, appropriate autophagy promotes disc cell survival. Autophagy helps disc cells scavenge ROS through self-digestion to protect disc cells from oxidative damage. However, cell death caused by excessive autophagy probably decreases the number of viable and functional cells in discs further [24, 85, 86]. Therefore, the dual roles of disc cell autophagy should be investigated in further researches.

### 6.3. ROS and Disc Cell Senescence

Cell senescence is an irreversible cell-cycle arrest resulting from DNA damage, telomere uncapping, oxidative stress, proinflammatory cytokines, and so forth. Senescent cells are viable and manifest a proinflammatory and catabolic phenotype defined as senescence-associated secretory phenotype (SASP). Previous studies have demonstrated the accumulation of senescent disc cells in human and rat degenerative discs [87, 88]. On the one hand, disc cell senescence promotes the loss of viable and functional disc cells due to replicative exhaustion. On the other hand, senescent disc cells secrete matrix proteases, cytokines, and chemokines to deteriorate the microenvironment of discs. Thus, cell senescence is a potential therapeutic target for IDD [89, 90]. However, the mechanism of disc cell senescence is very complex. ROS are an essential trigger of disc cell senescence. H_2_O_2_ resulted in a rapid increase in ROS production and DNA damage in human NP cells. Consequently, the ATM-Chk2-p53-p21-Rb pathway and the p16-Rb pathway were activated to induce premature senescence of human NP cells [15, 34]. H_2_O_2_ also induced premature senescence of human CEP cells through the p53-p21-Rb pathway [91]. Moreover, ROS overproduction induced by high glucose stress accelerated the senescence of rat AF and notochordal cells through the p16-Rb pathway [59, 60]. Taking these findings into account, recovering the redox homeostasis of disc cells is an effective measure to retard disc cells senescence.

### 6.4. ROS and Matrix Structure

The ECM of discs mainly comprises PGs and collagens that form a matrix network. This dynamic network is crucial to disc function as a shock absorber to resist mechanical loadings exerted on the spine. However, the structural failure of matrix network triggers IDD under abnormal mechanical loadings. As mentioned above, the structural components of disc matrix are vulnerable to oxidative damage. Posttranslational oxidative modification of the disc matrix components occurred during the process of IDD. The levels of AGEs and protein carbonylation increased significantly in human and mouse degenerative discs [74–78]. The oxidative modifications of collagens led to the crosslink and aggregation of collagens in discs and also induced the conformational changes of oxidized proteins, disrupting the primary, secondary, and triple-helical structure of collagens and causing collagen unfolding [78, 92]. As a consequence, the anatomic integrity and biomechanical property of disc matrix network altered [93].

### 6.5. ROS and ECM Metabolism

An imbalance between matrix anabolism and catabolism is an essential event during the process of IDD. Actually, the metabolism of ECM is tightly associated with the redox state in discs. Numerous studies have shown that H_2_O_2_ significantly downregulated the expression of collagen type II and aggrecan in human and rat disc cells [14, 15, 36, 38, 41, 44]. ROS overproduction induced by proinflammatory cytokines or high oxygen tension prominently suppressed the matrix synthesis and upregulated the expression of matrix degradation proteases in human and rat disc cells [14, 15, 21, 36, 44, 57]. ROS also caused a dramatic loss of PGs, collagens, and fibronectin in mouse discs [78]. Moreover, oxidized collagens in mouse discs were identified [78]. They are susceptive to proteolytic attack resulting from matrix proteases [94]. Generally, oxidative stress disturbs the balance between matrix anabolism and catabolism, resulting in a significant decrease in the matrix content of discs.

To sum up, oxidative stress induces the damage of matrix structure and promotes matrix degradation in IVDs. As a consequence, a significant loss of the elasticity and an increased stiffness of discs impair the mechanical function of discs, triggering IDD.

### 6.6. Comprehensive Effects of Oxidative Stress in the Establishment and Progression of IDD

Numerous lines of evidence indicate that ROS are widely involved in the signal transduction, metabolic regulation, programmed cell death, senescence, and phenotypic shift of disc cells. In fact, IDD is a disc cell-mediated pathological process. Disc degeneration is strongly associated with the viability and function of disc cells. Thus, ROS regulate the viability and function of disc cells affect the progression of IDD. Excessive ROS cause oxidative stress to activate various signaling pathways in disc cells, including the NF-*κ*B pathway and the MAPK pathway. Consequently, the phenotype of disc cells changes from a matrix anabolic phenotype into a matrix catabolic and proinflammatory phenotype. This phenotypic shift causes a dramatic matrix loss and enhances inflammation in the microenvironment of discs. Furthermore, chemokines secreted by disc cells recruit more immune cells into discs to enhance inflammation further. These immune cells secrete more cytokines and chemokines to deteriorate the viability and function of disc cells, forming a vicious circle [13].

Oxidative stress is a potent trigger of disc cell autophagy, apoptosis, and senescence. Autophagy provides recycled metabolic substrates to disc cells under oxidative stress, which protects disc cells from oxidative damage. However, excessive autophagy induced by sustained oxidative stress will lead to autophagic death of disc cells. Also, oxidative stress can directly induce disc cell apoptosis. Therefore, the number of viable and functional disc cells decreases markedly under sustained oxidative stress. More than that, this decrease cannot be compensated through cell proliferation due to disc cell senescence. More seriously, senescent disc cells secrete proinflammatory cytokines to promote the death or senescence of neighbouring disc cells, reinforcing the decrease in the number of viable and functional disc cells.

Degenerative discs show a significant structure failure. This is partially attributed to oxidative stress. ROS react with matrix components of discs, inducing oxidative modifications of matrix components. Modified components undergo structural changes, impairing the mechanical function of IVDs. As a result, IVDs gradually manifest degenerative changes under mechanical stimulation.

**Figure 3:**
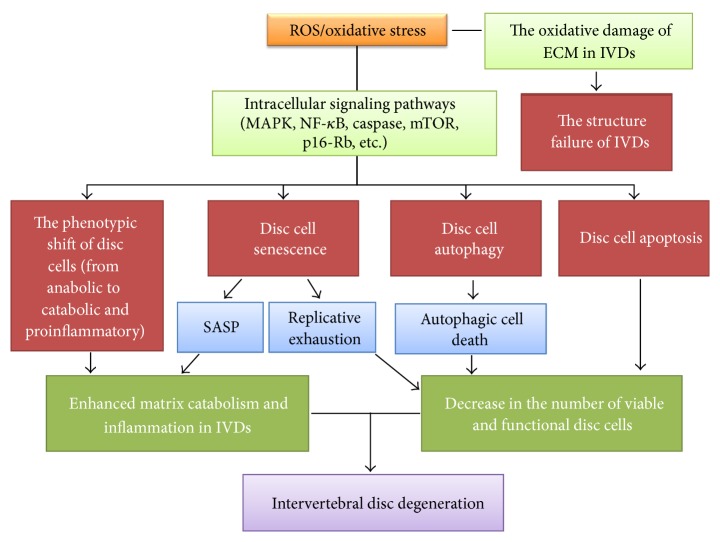
The involvement of reactive oxygen species (ROS)/oxidative stress in the pathogenesis of intervertebral disc (IVD) degeneration (IDD). ROS activate various signaling pathways in IVD cells and consequently regulate the phenotype, apoptosis, autophagy, and senescence of disc cells. Sustained oxidative stress induced by ROS overproduction reinforces matrix degradation and inflammation and enhances the decrease in the number of viable and functional disc cells in IVDs. Furthermore, ROS alter the extracellular matrix (ECM) structure of IVDs through oxidative modification, impairing the mechanical function of IVDs. As a result, the progression of IDD is accelerated. SASP: senescence-associated secretory phenotype.

In conclusion, oxidative stress plays a crucial role in the pathogenesis of IDD. It not only regulates the viability and function of disc cells, but also affects the ECM structure of discs. We illustrate the involvement of ROS/oxidative stress in the pathogenesis of IDD in Figure 3. Regulating the redox balance of disc cells to ameliorate oxidative stress is a promising therapeutic measure for IDD. Recent studies provide support to this idea. Broad complex-Tramtrack-Bric-a-brac and cap‘n'collar homology 1 deficient (Bach 1−/−) mice highly express heme oxygenase-1 (HO-1). HO-1 is an antioxidant enzyme that converts toxic hemes into antioxidants. It protects living cells from oxidative stress [95]. Notably, the expression of HO-1 in the punctured discs of Bach 1−/− mice was significantly higher than that in the punctured discs of wild-type mice. Concomitantly, the progression of IDD in Bach 1−/− mice was slower than that in wild-type mice [96]. AGEs were found to accumulate in the discs of diabetic mice or rats that manifest accelerated degenerative changes. However, the oral treatment of pyridoxamine (AGE inhibitor) ameliorated ROS production and inflammation in the discs of diabetic mice and consequently decelerated the progression of IDD [97, 98]. Moreover, the ERCC1-deficient mice manifest accelerated disc degeneration with aging due to DNA repair deficiency. However, the systematic treatment of XJB-5-131 (a mitochondrial-targeted ROS scavenger) potently delayed the progression of IDD in ERCC1-deficient mice [57]. Therefore, antioxidation is a new effective therapeutic strategy for IDD.

## 7. Therapeutic Implications

Oxidative stress is detrimental to the structural and functional homeostasis of discs. Thus, antioxidant supplementation is proposed as a promising measure for IDD treatment (Table 2). In this section, we will discuss the effect of various antioxidants on retarding the progression of disc degeneration.

### 7.1. Nonenzymatic Antioxidants

GSH is a major antioxidant in living cells. It was shown to reduce the IL-1*β*-induced ROS generation in human NP cells and to suppress the H_2_O_2_-induced apoptosis and matrix catabolism of human NP cells in vitro [44]. N-Acetylcysteine (NAC) is a precursor of GSH. NAC has been reported to decrease the level of ROS and consequently to suppress the activation of MAPK pathway and AMPK/mTOR pathway in human and rat disc cells in vitro [14, 15, 24, 25]. As a result, the inductive effect of ROS on the catabolic and proinflammatory phenotype of disc cells was suppressed. The autophagy and apoptosis of disc cells were attenuated. The premature senescence of disc cells caused by oxidative stress also was ameliorated [15, 23–25]. Moreover, the oral administration of NAC inhibited oxidative stress, matrix catabolism, and inflammation in rat discs to retard disc degeneration induced by needle puncture [14].

### 7.2. Polyphenols

Polyphenols are natural compounds found in vegetables, fruits, tea, wine, and chocolate. Their antioxidative and anti-inflammatory properties have been widely investigated [99, 100]. Resveratrol (RSV) is a polyphenol compound identified in plants. Previous studies have investigated the effects of RSV on human, rat, and bovine NP cells in vitro. RSV suppressed NP cell death as well as senescence and promoted NP cell proliferation through activating silent information regulator 2 ortholog 1 (SIRT1) and PI3K/Akt/caspase-3 pathway [26–32]. SIRT1 is a longevity gene. It stimulates the expression of antioxidants and suppresses the activation of NF-*κ*B pathway in cells. The activation of SIRT1 was shown to attenuate the H_2_O_2_-induced senescence of human CEP cells in vitro [91]. In addition, RSV suppressed the activation of various transcriptional factors in NP cells, including AP-1 and AP-2, CREB, Ets1/PEA3, E2F1, estrogen RE, NF-*κ*B, Sp1, and STATs. As a consequence, the PG synthesis of NP cells was enhanced. The expression of matrix proteases and cytokines by NP cells was downregulated [28, 31–33]. Noticeably, in vivo studies have documented that RSV upregulates the expression of aggrecan and SIRT1 and downregulates the expression of MMP-3 and p16 to retard the degeneration of rodent punctured discs [29]. Polyphenol epigallocatechin 3-gallate is a polyphenol redox scavenger. In vitro investigations have found that polyphenol epigallocatechin 3-gallate not only suppresses the senescence and apoptosis of human NP cells under oxidative stress, but also inhibits the expression of cytokines and MMPs in human NP cells through regulating the MAPK pathway and the NF-*κ*B pathway [34, 35].

### 7.3. ROS Scavengers

Pyrroloquinoline quinone (PQQ) is a critical cofactor of mitochondrial dehydrogenases and a ROS scavenger [101, 102]. For disc cells, PQQ suppressed the H_2_O_2_-induced ROS overproduction in rat NP cells and subsequently protected rat NP cells from H_2_O_2_-induced apoptosis in vitro. It also antagonized the downregulation of collagen type II and aggrecan in rat NP cells induced by H_2_O_2_ [41]. Fullerenes are powerful ROS scavengers due to their sustained activity, unique nanostructures, and great cell membrane-penetrating ability [103, 104]. Fullerol, a polyhydroxylated derivative of fullerenes, has a potent scavenging ability against ROS compared with SOD and mannitol. It was found to reduce ROS production in human NP cells in vitro. At the same time, it attenuated the upregulation of matrix proteases as well as the downregulation of collagen type II induced by H_2_O_2_ in cultured human NP cells. Moreover, intradiscal injection of fullerol protected the punctured rabbit discs from degeneration through promoting matrix synthesis and suppressing ectopic ossification [36].

### 7.4. Herbal Components

Ferulic acid (4-hydroxy-3-methoxy cinnamic acid, FA) is a phenolic antioxidant found in Chinese herb medicine. It has been reported to have anti-inflammation, antiapoptosis, anticancer, and antiaging properties [105, 106]. FA suppressed ROS accumulation in cultured rabbit NP cells and consequently retarded apoptosis. It also upregulated the expression of aggrecan and collagen type II and downregulated the expression of MMP-3 in cultured rabbit NP cells under oxidative stress [42, 43].* Cordyceps militaris* is a Chinese herb medicine. Cordycepin (3′-deoxyadenosine) is one of the bioactive components isolated from* Cordyceps militaris*. Recently, the anti-inflammatory, antiaging, antioxidative, and anticancer effects of cordycepin have been documented [107, 108]. Cordycepin suppressed the lipopolysaccharide- (LPS-) induced ROS production and NF-*κ*B pathway activation to prevent the LPS-induced phenotypic shift of rat NP cells from an anabolic phenotype to a catabolic phenotype. Furthermore, cordycepin protected organ-cultured rat IVDs from LPS-induced degeneration ex vivo [37].

### 7.5. Growth Factors

Several growth factors have been reported to protect disc cells from oxidative damage. Bone morphogenetic protein-7 inhibited the proapoptotic effect of H_2_O_2_ on human NP cells in vitro, which helps human NP cells maintain the ability of matrix synthesis under oxidative stress [38]. Insulin-like growth factor-1 ameliorated premature senescence of human AF cells induced by H_2_O_2_ in vitro [39]. Besides, hepatocyte growth factor protected rabbit NP cells from H_2_O_2_-induced apoptosis in vitro. It also downregulated the expression of matrix proteases and proinflammatory cytokines in rabbit NP cells [40].

## 8. Conclusion

The contributions of oxidative stress to the pathophysiology of IDD are complicated. More and more researchers devote themselves to elucidating the association between oxidative stress and disc degeneration. However, our knowledge of this issue is limited. Further investigations are required urgently. Antioxidative therapy is suggested as a promising therapeutic approach for IDD. Various antioxidants, such as NAC, food polyphenols, ROS scavengers, and growth factors, have been demonstrated to prevent the deleterious effects of ROS on disc cells in vitro (Table 2). However, there are not enough in vivo lines of evidence to support the effectiveness of antioxidants on preventing or retarding the establishment and progression of IDD. Furthermore, the effect of antioxidants on relieving IDD-associated LBP remains unknown. Thus, further studies based on in vivo preclinical studies and clinical studies will be needed to develop an effective antioxidative therapy for IDD.

## Figures and Tables

**Table 1:** Reactive oxygen species (ROS) sensitive signaling proteins in disc cells.

ROS sensitive signaling molecules	Experimental models	Cellular processes regulated by the molecules	Reference
ERK, JNK, and p38	Rat AF cells (in vitro)	Matrix metabolism Proinflammatory phenotype	[14]
ERK, JNK, p38, Akt, p65 Nrf2, ATM, Chk2, and p53	Human NP cells (in vitro)	Cell cycle progression Matrix metabolism Proinflammatory phenotype Antioxidative system	[15]
p65	Human NP cells (in vitro)	Proinflammatory phenotype	[20]
JNK and p38	Bovine NP cells (in vitro)	Matrix catabolic phenotype	[21]
ERK	Rat NP cells (in vitro)	Autophagy	[22]

AF: annulus fibrosus; NP: nucleus pulposus.

**Table 2:** Therapeutic effects of antioxidants on degenerative disc cells and intervertebral disc degeneration.

Antioxidant	Model (administration route)	Therapeutic effects	Reference
NAC	Rat AF cells (in vitro supplementation)	Suppressing catabolic and proinflammatory phenotype induced by H_2_O_2_	[14]
	Rat discs (oral administration)	Delaying IDD process induced by disc puncture	[14]
	Human NP cells (in vitro supplementation)	Retarding premature senescence induced by H_2_O_2_	[15]
	Rabbit AF cells (in vitro supplementation)	Restraining apoptosis induced by local anesthetics	[23]
	Rat NP cells (in vitro supplementation)	Suppressing excessive autophagy induced by serum deprivation	[24]
	Rat NP cells (in vitro supplementation)	Suppressing excessive autophagy induced by compression	[25]

Resveratrol	Human NP cells (in vitro supplementation)	Suppressing apoptosis	[26, 27]
	Bovine NP cells (in vitro supplementation)	Inhibiting matrix catabolic phenotype and promoting matrix anabolism	[28]
	Mouse discs (oral administration)	Delaying IDD process induced by disc puncture	[29]
	Human NP cells (in vitro supplementation)	Inhibiting matrix catabolic phenotype and promoting matrix anabolism	[30]
	Rat NP cells (in vitro supplementation)	Inhibiting apoptosis induced by IL-1*β*	[31]
	Human NP cells (in vitro supplementation)	Suppressing matrix catabolic phenotype induced by TNF-*α*	[32]
	Human NP cells (in vitro supplementation)	Suppressing proinflammatory phenotype induced by IL-1*β*	[33]

EGCG	Human NP cells (in vitro supplementation)	Retarding premature senescence induced by H_2_O_2_	[34]
	Human NP cells (in vitro supplementation)	Suppressing the proinflammatory and catabolic phenotype induced IL-1*β*	[35]

Fullerol	Human NP cells (in vitro supplementation)	Retarding matrix catabolism induced by H_2_O_2_	[36]
	Rabbit discs (intradiscal injection)	Delaying IDD process induced by disc puncture	

Cordycepin	Rat NP cells (in vitro supplementation)	Suppressing matrix catabolic phenotype induced by LPS	[37]
	Rat organ cultured discs (ex vivo supplementation)	Delaying IDD process induced by LPS	

BMP7	Human NP cells (in vitro supplementation)	Suppressing apoptosis and matrix catabolic phenotype induced by H_2_O_2_	[38]
IGF1	Human AF cells (in vitro supplementation)	Retarding premature senescence induced by H_2_O_2_	[39]
HGF	Rabbit NP cells (in vitro supplementation)	Suppressing apoptosis and matrix catabolic and proinflammatory phenotype induced by H_2_O_2_	[40]
PQQ	Rat NP cells (in vitro supplementation)	Suppressing apoptosis and matrix catabolic phenotype induced by H_2_O_2_	[41]
Ferulic acid	Rabbit NP cells (in vitro supplementation)	Restraining apoptosis and matrix catabolic phenotype by H_2_O_2_	[42, 43]
GSH	Human NP cells (in vitro supplementation)	Suppressing apoptosis and matrix catabolic phenotype induced by H_2_O_2_	[44]

NP: nucleus pulposus; AF: annulus fibrosus; GSH: glutathione; NAC: N-acetylcysteine; IDD: intervertebral disc degeneration; IL: interleukin; TNF: tumor necrosis factor; EGCG: epigallocatechin 3-gallate; PQQ: pyrroloquinoline quinone; LPS: lipopolysaccharide; BMP: bone morphogenetic protein; IGF: insulin-like growth factor; HGF: hepatocyte growth factor.

## Abbreviations

ROS: Reactive oxygen species
IVD: Intervertebral disc
IDD: Intervertebral disc degeneration
LBP: Low back pain
CEP: Cartilage endplate
PG: Proteoglycan
NP: Nucleus pulposus
AF: Annulus fibrosus
SOD: Superoxide dismutase
ECM: Extracellular matrix
MAPKs: Mitogen-activated protein kinases
NO: Nitric oxide
PKC: Protein kinase C
TNF: Tumor necrosis factor
MMP: Matrix metalloproteinase
NOX: Nicotinamide adenine dinucleotide phosphate (NADPH) oxidase
XO: Xanthine oxidase
GSH: Glutathione
MPTP: Mitochondrial permeability transition pore
MTMP: Mitochondrial membrane potential
Msr: Methionine sulfoxide reductase
MDA: Malondialdehyde
AGEs: Advanced glycation end products
CML: Carboxymethyl-lysine
AOPP: Advanced oxidation protein products
SASP: Senescence-associated secretory phenotype
NAC: N-Acetylcysteine
RSV: Resveratrol
SIRT1: Silent information regulator 2 ortholog 1
PQQ: Pyrroloquinoline quinone
FA: Ferulic acid
COX: Cyclooxygenase.
